# Hybrid ^18^F-FDG-PET/MRI Measurement of Standardized Uptake Value Coupled with Yin Yang 1 Signature in Metastatic Breast Cancer. A Preliminary Study

**DOI:** 10.3390/cancers11101444

**Published:** 2019-09-26

**Authors:** Concetta Schiano, Monica Franzese, Katia Pane, Nunzia Garbino, Andrea Soricelli, Marco Salvatore, Filomena de Nigris, Claudio Napoli

**Affiliations:** 1 IRCCS SDN, 80134 Naples, Italy; 2 Department of Motor Sciences and Healthiness, University of Naples Parthenope, 80134 Naples, Italy; 3 Department of Precision Medicine, University of Campania “Luigi Vanvitelli”, 80138 Naples, Italy; 4 Department of Advanced Medical and Surgical Sciences, University of Campania “Luigi Vanvitelli”, 80138 Naples, Italy

**Keywords:** breast, imaging, marker, radiomics, Yin Yang 1

## Abstract

Purpose: Detection of breast cancer (BC) metastasis at the early stage is important for the assessment of BC progression status. Image analysis represents a valuable tool for the management of oncological patients. Our preliminary study combined imaging parameters from hybrid ^18^F-FDG-PET/MRI and the expression level of the transcriptional factor Yin Yang 1 (YY1) for the detection of early metastases. Methods: The study enrolled suspected *n* = 217 BC patients that underwent ^18^F-FDG-PET/MRI scans. The analysis retrospectively included *n* = 55 subjects. *n* = 40 were BC patients and *n* = 15 imaging-negative female individuals were healthy subjects (HS). Standard radiomics parameters were extracted from PET/MRI image. RNA was obtained from peripheral blood mononuclear cells and YY1 expression level was evaluated by real time reverse transcription polymerase chain reactions (qRT-PCR). An enzyme-linked immuosorbent assay (ELISA) was used to determine the amount of YY1 serum protein. Statistical comparison between subgroups was evaluated by Mann-Whitney U and Spearman’s tests. Results: Radiomics showed a significant positive correlation between Greg-level co-occurrence matrix (GLCM) and standardized uptake value maximum (SUVmax) (*r* = 0.8 and *r* = 0.8 respectively) in BC patients. YY1 level was significant overexpressed in estrogen receptor (ER)-positive/progesteron receptor-positive/human epidermal growth factor receptor2-negative (ER+/PR+/HER2-) subtype of BC patients with synchronous metastasis (SM) at primary diagnosis compared to metachronous metastasis (MM) and HS (*p* < 0.001) and correlating significantly with ^18^F-FDG-uptake parameter (SUVmax) (*r* = 0.48). Conclusions: The combination of functional ^18^F-FDG-PET/MRI parameters and molecular determination of YY1 could represent a novel integrated approach to predict synchronous metastatic disease with more accuracy than ^18^F-FDG-PET/MRI alone.

## 1. Introduction

PET and MRI scans are useful instruments to diagnose the presence of metastatic disease providing both morphological and metabolic information [1,2,3]. However, an exclusive use of imaging technique, has some limitations, such as high cost, sensitivity and specificity [4]. We are confident that the combination of non-invasive methods with the parameters derived from image analysis will be of great interest for clinical practice.

A growing amount of data has established that polycomb oncoprotein Yin Yang 1 (YY1) has a prognostic impact in malignancy [5,6,7]. YY1 is frequently overexpressed in a wide range of solid and non-solid tumors, regulating a broad class of genes involved in control of both cell cycle and apoptosis. In vitro and in vivo studies have reported the expression of YY1 has been associated with development of a malignant phenotype in some human cancers, tumor progression, metastasis formation, and correlating with poor prognosis and drug/immune resistance [8,9,10,11,12,13,14,15,16,17]. However, conflicting results have been shown when YY1 was evaluated during the progression and metastasis of breast cancer (BC). The role of YY1 in metastatic BC has recently been verified by meta-analysis showing that YY1 exhibited significantly high mRNA levels in basal-like BCs versus normal tissues [18]. In contrast, Lieberthal et al. reported an increased expression of YY1 in invasive BC cells [19]. Furthermore, recently, YY1 was reported as anti-oncogene in invasive human BC cells [20]. Specifically, three potential binding sites for YY1 were found on a lincRNA involved in occurrence and progression of human cancers [20]. Moreover, about 70%–80% of diagnosed BCs are estrogen receptor positive and treated with endocrine therapy. However, this treatment is ineffective for patients with metastatic disease [21].

This study represents the first assessment of YY1 mRNA and protein expression levels in peripheral blood from BC patients, which have been correlated with imaging parameters from hybrid ^18^F-FDG-PET/MRI in order to improve the early detection of metastatic disease.

## 2. Materials and Methods

### 2.1. Study Cohort 

**Figure 1:**
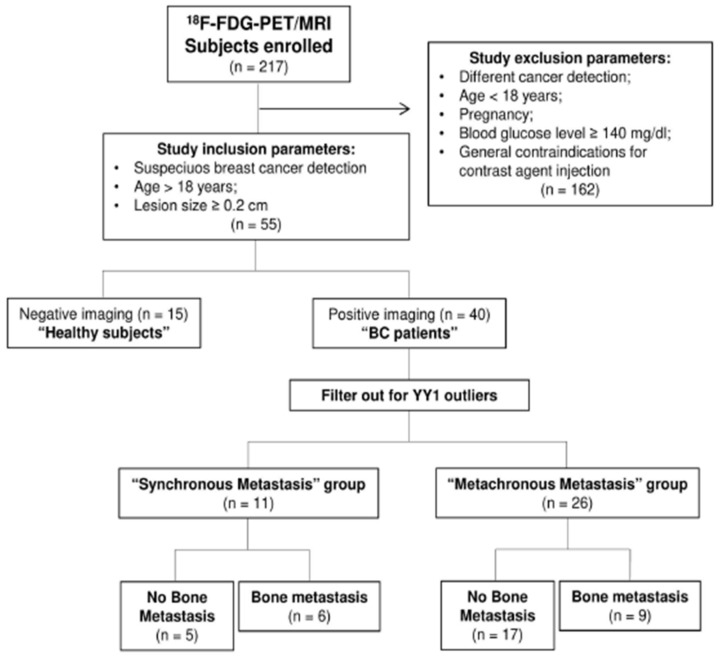
Eligible patients included in the study. Subject flow chart describing inclusion and exclusion criteria for the selection of the patients.

The study was approved by the institutional ethics committee (IRCCS Fondazione SDN) in accordance with the ethical standard of the Declaration of Helsinki. A written informed consent was obtained from all subjects enrolled (healthy subjects (HS) and BC patients). All clinical-pathological characteristics are reported in Table 1. Exclusion criteria included age <18 years, pregnancy, different cancer detection, and general contraindications for contrast agent injection. All eligible patients had ^18^F-FDG-PET/MRI scans between 2014 and 2018. Our retrospective study enrolled consecutive suspected *n* = 217 oncological patients that underwent ^18^F-FDG-PET/MRI scans. Individuals with negative imaging were considered HS (*n* = 15) (Figure 1) [22]. 

### 2.2. Sample Collection

Peripheral blood mononuclear cells (PBMCs) and serum aliquots were isolated as previously described [23]. Briefly, PBMCs were isolated by density gradient centrifugation on Histopaque-10771 (Sigma-Aldrich). After centrifugation, white blood cells were recovered and washed twice with phosphate-buffered saline (PBS) pH7.4 (Gibco; Life Technologies/Gibco, Italy). White cells and serum aliquots were frozen at −80 °C at the IRCCS SDN Biobank [24].

### 2.3. RNA Extraction and Real-Time Quantitative Reverse Transcription Polymerase Chain Reaction (qRT-PCR) Assay

Total RNAs were extracted using Trizol solution (Life Technologies/Gibco, Italy), according to the manufacturer’s instructions. The specificity of oligonucleotide pair used was verified with the Basic Local Alignment Search Tool (BLAST), NCBI program and through in-silico polymerase chain reaction (PCR) analysis by UCSC (Genome Browser website) [23]. Primers were designed by Primer 3 software (http://bioinfo.ut.ee/primer3-0.4.0/) and synthesized by Life Technologies [23]. Quantitative real-time PCR was performed according to the supplier protocols (BioRad). Melt curve analysis was performed to verify a single product species. Selective primer sequences used for YY1 (NM_003403) were 5′-CAAAACTAAAACGACACCAAC-3′ (Forward) and 5′-TGAAGTCCAGTGAAAAGCGT-3′ (Reverse). Ribosomal protein S18 (RPS18; NM_022551) was used as housekeeping gene [23]. All reactions were carried out at least in triplicate for every cDNA template and qRT-PCR data were analyzed by Ct (threshold cycle) approach. The expression levels of the target gene normalized with the internal housekeeping gene were presented as delta-Ct (ΔCt), calculation [23]. The relative expression was estimated among groups considered.

### 2.4. YY1 Enzyme-Linked Immunosorbent Assay (ELISA) Test

Prior analysis, serum samples were stored at -80 °C. All samples were thawed only once prior to use. For the detection and quantification of our antigen of interest, enzyme-linked immunosorbent assay (ELISA) was used, according to the manufacturer’s instructions with signals detection at 450 nm (Biocompare, Italy Cat. number ABIN4973888). Kit detection range was 0.156–10 ng/mL with sensitivity <0.059 ng/mL.

### 2.5. Hybrid ^18^F-FDG-PET/MRI Technique

All patients were acquired on both a PET/CT device and on a 3T hybrid PET/MRI system (mMR Biograph; Siemens, Erlangen, Germany, with system sensitivity of 13.2 cps/kBq, transverse resolution of 4.4 mm and axial resolution of 4.5 mm) equipped with a dedicated breast coil, according to previously described acquisition protocols [25,26]. After fasting for 8 hours, patients were administered about 401 ± 32 MBq (mean ± standard deviation) of ^18^F-FDG was administered to the patients, through an antecubital catheter. ^18^F-FDG-PET/MRI images were performed concurrently after an uptake period of more than 60 minutes. The protocol was optimized for accurate detection and staging as previously described [25,27], briefly: an axial T2 turbo spin echo sequence; axial and coronal T2 turbo inversion recovery; diffusion weighted imaging (DWI), considered the most specific technique for the breast study. To investigate both morphological and dynamic breast parameters, all patients were injected with gadolinium diethylene triamine pentacetate (GD-DTPA; Magnevist) at 0.1 mmol/kg body weight, The dynamic study provides six axial spoiled gradient echo (SPGRE) 3D VIBE sequence before, during and after injection of contrast agent with variable flip-angle thus making a fat suppression. The attenuation correction is given by the segmentation (DIXON) in tissue classes, obtaining both water and fat maps. PET data are related only to these specific elements. Considering the PET data, the process of image reconstruction is done through an iterative algorithm called OSEM composed by three iterations on a matrix 172 × 172. Its main feature is the noise reduction in regions of low absorption. Image noise control is obtained by dividing data into 21 subsets analyzed cyclically. 

### 2.6. Image Analysis

**Figure 2:**
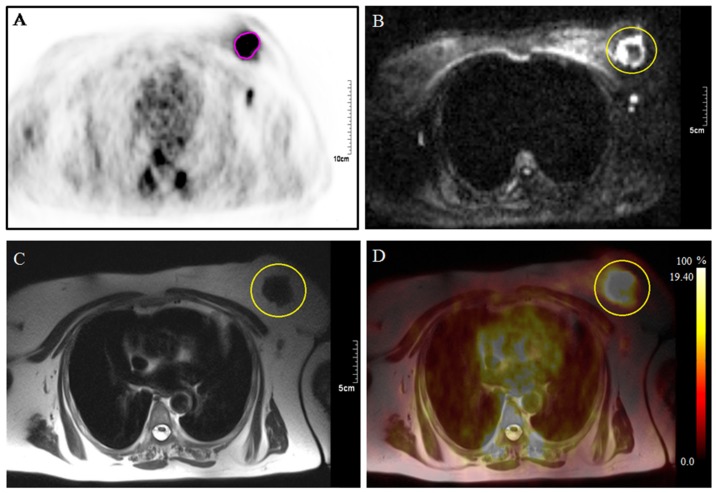
^18^F-FDG-PET/MRI scan Images of a 57-year-old patient with a breast heteroplasia with lymph node and bone metastases. The yellow circle indicates the lesion located on the left breast: (**A**) PET image showing uptake after 60 minutes of a segmented lesion (pink depicted); (**B**) Lesion revealed on T2 weighted image acquired on the axial plane; (**C**) hyperintensity of the lesion on diffusion-weighted magnetic resonance imaging; (**D**) ^18^F-FDG-PET/MRI fusion image.

This study enrolled consecutive n. 217 subjects, of whom n. 15 HS and n. 202 oncological patients that underwent PET/MRI scans (Figure 1). After screening criteria, n. 52 female subjects were retrospectively available for analysis; in detail, 37 were BC patients and 15 HS (Figure 1). BC primary lesions were classified according to the American Joint Committee on Cancer Disease (AJCC) [28]. BC histology was confirmed by immunohistochemistry (IHC) analysis only for few patients diagnosed. Quantitative analysis was performed using PMOD (Version 3.8; PMOD Technologies, Switzerland), which consists of a complete set of tools, able to extrapolate quantitative parameters from the image. After importing all the ^18^F-FDG-PET/MRI examinations into the PMOD software, the breast lesion automatic segmentation was done on the PET image. A reference cut-off of 41% of the standardized uptake value maximum (SUVmax) and volume of interest (VOI) were reported also on MRI images. Axial T2, DWI, apparent diffusion coefficient (ADC) and post administration 3D VIBE sequence were analyzed. One representative patient case was shown in Figure 2. For all subjects, we considered the following parameters: VOI, SUVmax and standardized uptake value minimum (SUVmin), average, standard deviation (SD), number of pixels and hot average (mean of pixels that presents higher values) of SUV body-weighted (SUVbw) and of SUV lean body mass (SUVlbm). SUVbw was expressed like c(t)/(Injected dose/body weight), where c(t) is tissue radioactivity concentration at time t. While SUVlbm referred to the value of standardized lean body mass absorption. Some texture indices referred to the histogram pixel within a VOI, and others based on to the gray-level co-occurrence matrix (GLCM). This latter matrix is defined as the distribution of co-occurring pixel values (grayscale values, or colors) at a given offset. In this stud, texture indices were: GLCM contrast (measure of the contrast intensity between pixels and its close over the entire image); Statistic Energy (the sum of the squared elements in the GLCM); GLCM sum variance (heterogeneity measure on close pixels).

### 2.7. Statistical Analysis

Statistical analyses were performed using R environment (http://www.R-project.org). The distribution of biomedical imaging data (PET and MRI) was tested by Shapiro–Wilk test normality. The correlation between the statistical and textural parameters mostly used for breast lesions was evaluated using (Spearman’s correlation test). In addition, the Wilcoxon–Mann–Whitney test was used to establish the statistical significance of molecular signature between the different BC groups and HS. To compare different hormone receptor subtypes, Kruskal–Wallis test was performed. For all tests a *p* value was considered for statistical significance when *p* ≤ 0.05 and most significant when *p* ≤ 0.01. Spearman’s correlation was calculated to compare values of SUV max and YY1 levels.

## 3. Results

### 3.1. Study Population and Imaging Features

The mean age of all subject enrolled was 50 ± 4.6 years in HS compared to 53 ± 2.8 years in BC patients. 

Moreover, within the metastatic BC cancer patients, we had patients with primary metastases (synchronous disease) and patients who developed metastases during follow-up (metachronous disease),.Thus, the cohort was divided in three groups: (*i*) “Healthy Subjects” (HS) (*n* = 15); (*ii*) “Synchronous Metastasis” (SM), which includes BC patients with one or multiple metastases at primary diagnosis (*n* = 11); and (*iii*) “Metachronous Metastasis” (MM), including BC patients who developed metastasis at follow up (*n* = 26). The metastasis were localized in bone (*n* = 9) and in different site, such as liver, uterus, lung, thyroid, and axillar and thoracic lymph nodes (*n* = 17). 

Several features were extrapolated in order to investigate the sensitivity, specificity, and accuracy of ^18^F-FDG-PET/MRI technique. By integration of high sensitivity of PET with the high spatial and temporal resolution of MRI we obtained information and characterized on the tumor malignancy (Figure 2) and detect small metastasis present in the body (Tumor Volume ≥1.2 cm^3^ and GLCM_contrast_T2 ≥ 0.002). All features were related to the media pixel histogram within a VOI and to the GLCM, as highlighted by PET and MRI images as reported in Table 2 and Table 3. 

### 3.2. YY1 Molecular Expression in Breast Cancer (BC) Patient 

**Figure 3:**
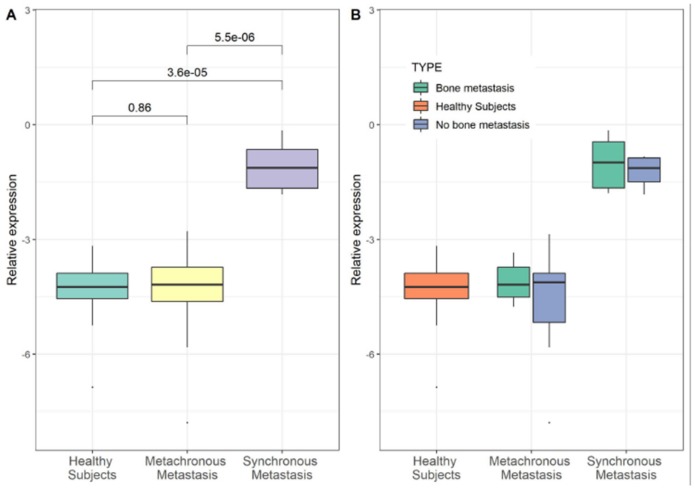
YY1 mRNA level and metastatic breast cancer. Expression level of YY1 in BC patients clustered into “Healthy Subjects” (HS), “Metachronous Metastasis” (MM) and “Synchronous Metastasis” (SM) group. (**A**) YY1 mRNA level in PBMCs from BC patients (*n* = 11 SM and *n* = 26 MM) and HS patients (*n* = 15). Data are reported as (-ΔCt). Each group was compared. (**B**) BC patients for SM and MM group were classified in two categories: no bone metastasis (*n* = SM and *n* = MM, respectively) and bone metastasis (*n* = 5 SM and *n* = 17 MM, respectively); the distribution did not show statistically significant difference between two categories included in each group. Statistical analysis was performed by the Wilcoxon test, and *p* value < 0.05 was considered significant.

**Figure 4:**
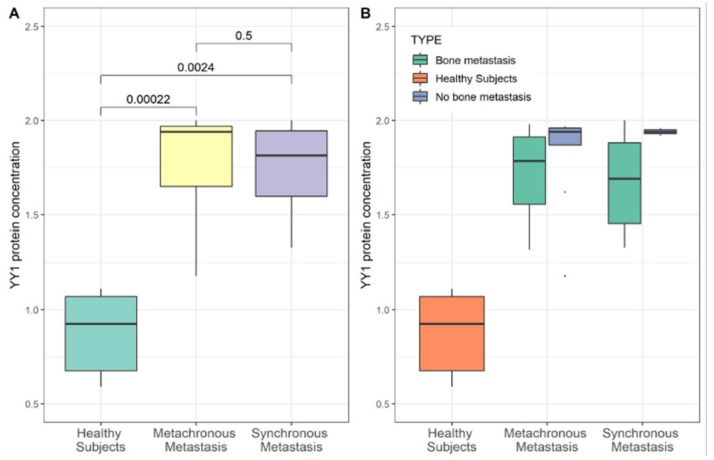
YY1 protein level and metastatic breast cancer. Serum protein level of YY1 in BC patients grouped as “Healthy Subjects” (HS), “Metachronous Metastasis” (MM) and “Synchronous Metastasis” (SM) group. (**A**) Comparison of YY1 protein level between BC patients (*n* = 11 SM and *n* = 26 MM) and HS patients (*n* = 15). Protein concentration is reported as ng/mL. Enzyme-linked immunosorbent assay (ELISA) YY1 protein level in BC patient sera indicated a media concentration of 1.8 ± 0.3 ng/ml in MM and 1.7 ± 0.3 ng/ml in SM patients compared to 0.8 ± 0.3 ng/ml in HS (*p* < 0.01; *p* < 0.01). (**B**) Comparison of YY1 protein level in SM and MM patients subgrouped as: no bone metastasis (*n* = 5 SM and *n* = 17 MM, respectively) and bone metastasis (*n* = 5 SM and *n* = 17 MM, respectively); Statistical significance was determined by Wilcoxon test, *p* value < 0.05 was considered significant.

In order to analyze the expression level of YY1, from all patients PBMCs were recovered and total RNAs were extracted. Gene YY1 expression was evaluated in BC patients compared to control samples by real time RT-PCR. Same amount of YY1 has been shown in the MM group compared HS group. Conversely, in the SM group showed a 20-fold increase of YY1 mRNA compared with the HS group and 19-fold increase compared with the MM group (*p* ≤ 0.01) using the non-parametric Wilcoxon test (Figure 3A). Moreover, the expression levels of YY1 (mRNA) were compared between MM and SM groups also considering the bone and no bone metastasis, respectively (Figure 3B). In contrast, YY1 protein level, dosed with ELISA assay, showed a significant increase in BC patients (MM and SM respectively, *p* < 0.01; *p* < 0.01). Differently from the expression trend, protein level of YY1 marker was higher into MM compared with HS group, although we did not know the underlying mechanism (Figure 4A,B).

**Figure 5:**
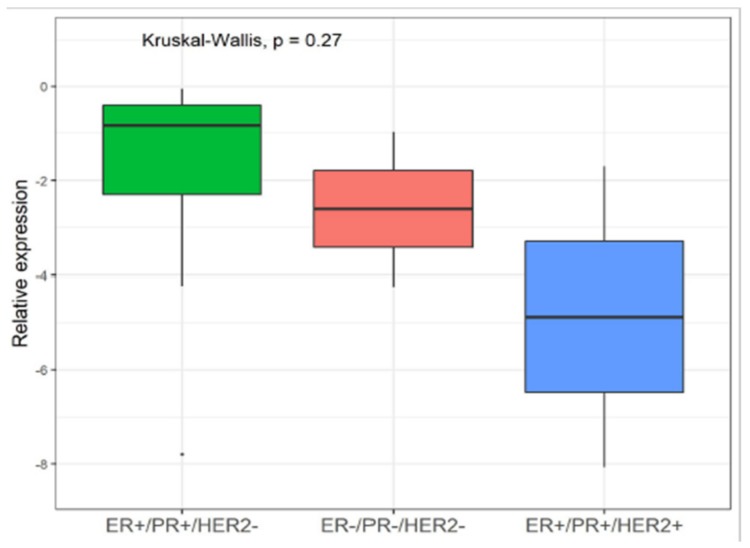
YY1 levels and receptor status in metastatic breast cancer. Comparison of YY1 mRNA level between estrogen receptor -positive/progesteron receptor-positive/human epidermal growth factor receptor2-negative (ER+/PR+/HER2-) compared to the “triple positive” (ER+/PR+/HER2+) and “triple negative” (ER-/PR-/HER2-) BC receptor status. Relative expression is reported as (-ΔCt). Statistical significance was determined by Kruskal-Wallis test, *p* value <0.05 was considered significant.

Up-regulation of YY1 has been shown in estrogen receptor-positive (ER+) BCs [29] however, the regulatory mechanisms are still unknown. In our preliminary analysis, we also found YY1 overexpression in ER+ compared to ER- tumors, taking into account a total of *n* = 15 patients (see Table 1). Interestingly, we observed that YY1 mRNA specifically increased in (ER)-positive/- progesteron receptor-positive (PR+)/-human epidermal growth factor receptor2-negative (HER2-) (ER+/PR+/HER2-) compared to the “triple negative” (ER-/PR-/HER2-) and “triple positive” (ER+/PR+/HER2+) BC subtypes (*p* = 0.27, Kruskal-Wallis test) (Figure 5). Therefore, these data needs to be further elucidated, taking into account larger cohort including also different HER2+ and HER2- combinations such as ER-/PR-/HER2+, not diagnosed in our cases.

### 3.3. Correlation Between YY1 and FDG-PET/MRI in BC Patients 

**Figure 6:**
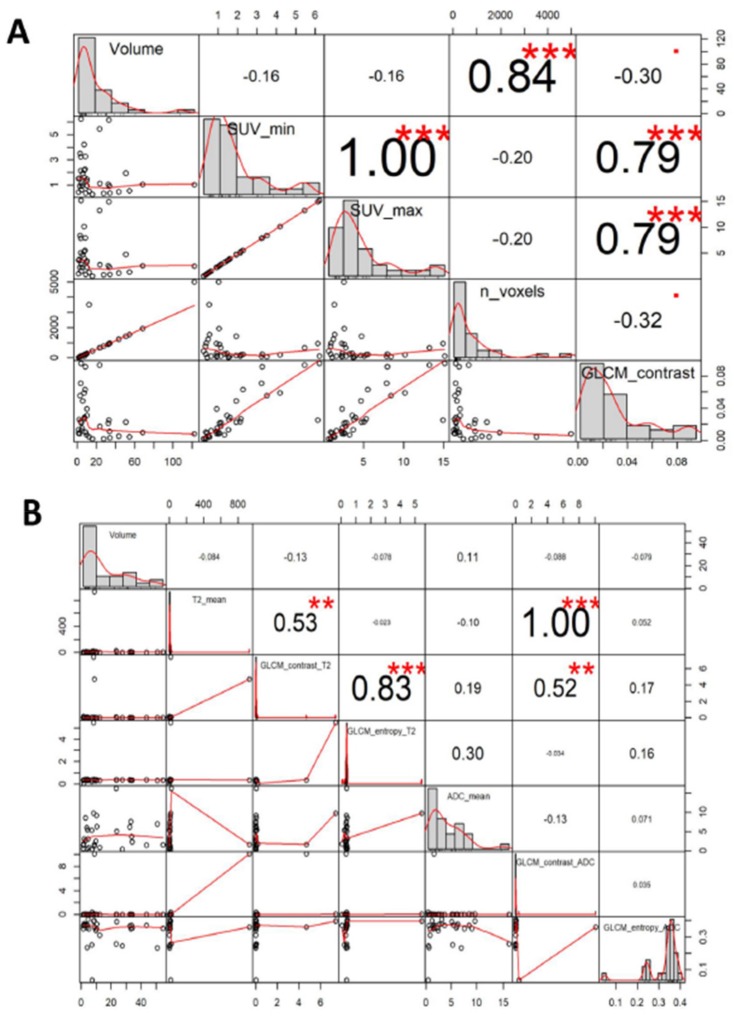
Correlation matrix for radiomics features of PET and MRI images. Correlation charts represent the correlation matrix between the statistical and textural parameters extracted from PET images (**A**) and MRI images (**B**) mostly used for breast lesions. The distribution of each variable is shown on the diagonal. On the bottom of the diagonal: the bivariate scatter plots with a fitted line are displayed. On the top of the diagonal: the value of the correlation plus the significance level as stars. Each significance level is associated to a symbol: *p* values 0.001; 0.01; 0.05; with symbols “***”; “**”; “*” respectively.

**Figure 7:**
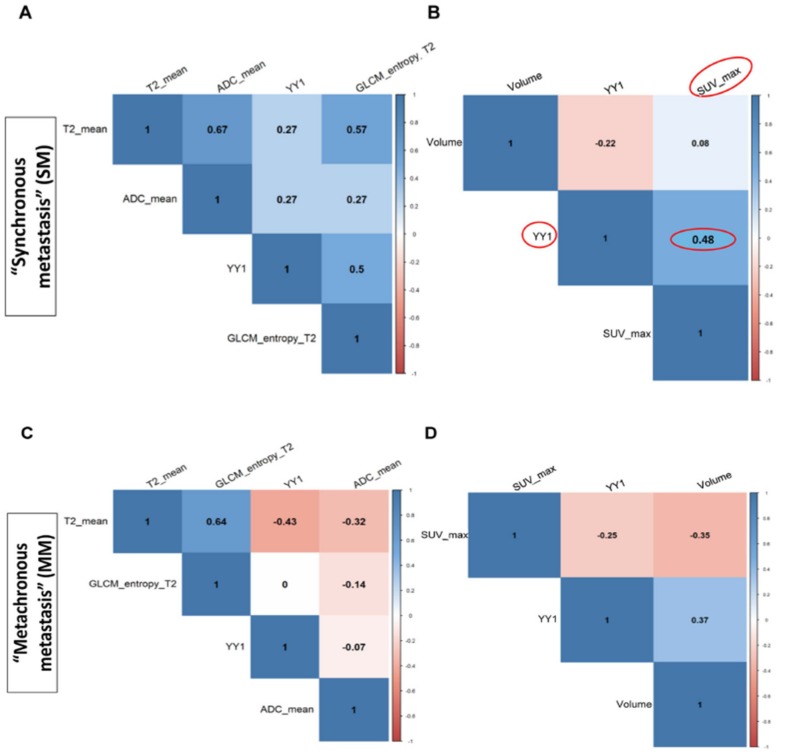
Radiomic features and YY1 molecular analysis. Correlation plot illustrates the Spearman test coefficient between YY1 expression level and PET/RMI parameters across “Synchronous Metatstasis” (SM) (**A**,**B**) and “Metachronous Metatstasis” (MM) BC patients (**C**,**D**). Color scale bar shows the positive correlation in blue and the negative correlation in pink; color intensity represents the strength of the correlation coefficient. Data indicated a statistical significant correlation between YY1mRNA and SUVmax in patients with SM ρ = 0.48. In the Supplementary Materials Table S1 are reported all analyzed findings for each patient at primary diagnosis.

In order to select ^18^F-FDG-PET/MRI parameters to correlate with YY1, first we evaluated the association between all imaging features by Spearman correlation test [30]. As shown in Figure 6, a significant positive correlation was observed between textural parameters: GLCM_contrast, SUVmin and SUVmax (*r* = 0.8; *r* = 0.8 respectively) (Table 2, Table 3 and Supplementary Materials Table S1) [30]. Subsequently, the Spearman test significantly correlated YY1 mRNA level (-ΔCt values) and ^18^F-FDG-uptake (SUVmax) (*r* = 0.48) in SM patients with invasive BC cancer (Figure 7A,B). As reported in Figure 7B, pink color indicates that there was a negative correlation between YY1 expression reported as ΔCt values and ^18^F-FDG-uptake. However, considering that small ΔCt values correspond to high gene expression, our preliminary results showed that YY1 mRNA expression and SUVmax reported the same trend (Figure 7B). Instead, no correlation was observed between YY1 protein and ^18^F-FDG-PET/MRI parameters (data not shown). Overall, our study is the first attempts to evaluate the potential of combining imaging differences between SM and MM breast patient subgroups and YY1 gene expression, for a better diagnostic analysis of tumor metastasis (see Discussion).

## 4. Discussion and Conclusions

This preliminary study evaluated the feasibility of using diagnostic imaging and blood circulating level of YY1 as prospective tool to assess the presence of metastasis and tumor grade at diagnosis. The combination of imaging parameters and molecular biomarkers to improve the sensitive and specificity of primary tumor and metastatic lesions diagnosis it is of great interest for both better clinical decision-making and treatment management. To date, an accurate diagnosis of BC patients is difficult due to the tumor heterogeneity of clinical presentation and the co-presence of almost undetectable micro-metastasis. Moreover, based on the expression of hormone receptors in tumor tissues, approximately 70%–80% of BC is ER+ [31,32,33,34]. 

The multifunctional factor YY1 is overexpressed in many types of cancer, including BC [14,35,36,37,38,39,40] with potential clinical significance [6,7,8,9,10], although Lieberthal et al. reported that a decreased YY1 expression might contribute to the invasive phenotype of metastatic BC cells [19]. However, other authors also previously reported that the YY1 protein level is lower in BC tissue than in the normal breast tissues [29,41,42]. Clearly, more intensive studies are needed to clarify the role of YY1 and its molecular mechanism in BC. Our preliminary data indicated that YY1 transcript in PBMCs of BC subjects showed a significant increase in patients with metastasis at primary diagnosis than the “Healthy Subjects” group. We indicated this group as “Synchronous Metastasis”. Interestingly, YY1 was particularly higher in patients BC with bone metastasis although not statistically significant. 

In our analysis, we included SM patients with specific BC receptor status; indeed our cohort included about 80% of ER+ tumors, which reflects the recurrence of the majority of breast cancer. As well-known, hormone receptor status is crucial for medical decision-making since strongly related to hormone therapy response. Preliminary data indicated that YY1 might play an important role in ER+ compared to ER- tumors in according to other receptors. Prompted by Lieberthal et al. results on BC model cell lines [19] we evaluated YY1 mRNA level in PBMCs from patients stratified as ER/PR-positive tumors with respect to triple negative (ER-/PR-/HER2-) and triple positive receptor status (ER+/PR+/HER2+). In particular, we observed that YY1 mRNA specifically increased in hormone receptor positive BCs (ER+/PR+/HER2-) compared to the “triple positive” (ER+/PR+/HER2+) and “triple negative” (ER-/PR-/HER2-) subtypes although not statistically significant. 

^18^F-FDG-PET is the gold standard for in vivo evaluation of tumor glucose metabolism and ^18^F-FDG uptake. In particular, post-contrast MR images were highly correlated to the molecular subtypes of BC, such as normal-like, luminal A and B, HER2-enriched, and basal-like [43]. Additionally, several radiomics features, such as GLCM textural features and SUVs are currently used to classify BC tumor grade [44,45,46]. In particular, SUVmax is common feature of ^18^F-FDG-PET that potentially reflects the tumor biology, prognosis and response to treatment [44,45,46]. Here, we reported for the first time, a correlation of ^18^F-FDG-PET parameters with YY1 molecular marker. We showed that SUVmax were statistically correlated with blood circulating YY1 mRNAs in a small number of BC patients (*r* = 0.48), suggesting that at higher tumor uptake correspond to high blood level of YY1 (mRNAs). In summary, our study attempts to include another layer of information, which is imaging “phenotype” to tumor metastasis diagnosis. However, imaging phenotype differences between the BC synchronous (SM) and metachronous metastasis (MM) groups, have been so far poorly investigated [47,48,49]. We assessed if the addition of imaging layer could contribute to better discriminate SM and MM BC subgroups in combination with YY1 gene expression.

Our results, showed a quite weak correlation between imaging and molecular features, although other information could be useful for further consideration. For instance, we found a positive correlation between YY1 expression (mRNA) and SUVmax for SM rather than MM subgroups. SUVmax is routinely used imaging diagnostic parameter [50]. In BC studies, it usually indicates the degree of tumor metabolism and hence can predict its behavior and prognosis [51]. 

In spite of our observations, the pathogenic role, the release mechanism and cell origin of circulating YY1 in blood remain unknown. 

Furthermore, the present study has some limitations, which probably affected also our correlation analysis. It was a retrospective study performed at a single institution with a relatively small number of patients, and recurrence was documented in very few subjects. Moreover, we were unable to analyze overall survival because there were no disease-related deaths among the study population. In detail, in our study, the ROI segmentation was performed automatically. Maybe a manual segmentation could provide more accurate data. Furthermore, the knowledge of the tumor status for each patient would allow a potential correlation between YY1 with the invasiveness of BC. Surely, a larger number of subjects with the same characteristics could improve data sensitivity and specificity. In addition, the data could not be validated internally. Finally, an external validation of our findings would allow us to generalize the results and discriminate YY1 trend in all BC subtypes.

Nevertheless, although there are all the previous limitations and further multicenter studies are needed to confirm our results, all the preliminary data reported above are promising and suggest that a combined approach between different technologies for the clinical diagnosis of BC can provide more differentiated information related to the detection of metastases for treatment decision.

## Figures and Tables

**Table 1:** Patient clinical-pathological characteristics.

	Number or Values
**Total Cohort**	52
Healthy Subjects “Control group”	15
Breast cancer group “Synchronous metastasis” (SM)	11
Breast cancer group “Metachronous metastasis” (MM)	26
Mean age (years)	57
Range (min–max)	32–88
**TNM**	13
T0	1
T1	2
T2	7
T3	3
NA	24
**Tumor Grade**	10
G2	8
G3	2
NA	27
**Ki 67 Status ^a^**	12
Low	4
High	8
NA	25
**ER Status**	15
Negative	3
Positive	12
NA	22
**PR Status**	15
Negative	3
Positive	12
NA	22
**Her2 Status ^b^**	13
Negative	9
Positive	3
NA	24

^a^ Ki67 ≥ 20% was considered High. ^b^ one Her2 Status was equivocal. NA = Not available.

**Table 2:** Descriptive statistics of features extracted from PET images.

**Synchronous Metastasis Group**					
***Features***	**Mean ± SD**	**Median**	**Min**	**Max**	**Range**
**Volume**	9.54 ± 7.06	8.73	1.91	23.30	21.39
**SUV_max**	5.16 ± 4.19	3.78	1.33	13.24	11.91
**n_voxels**	721.86 ± 1247.68	247.00	54.00	3516.00	3462.00
**Statistic_Energy**	1.01 ± 0.01	0.01	0.01	0.01	0.00
**GLCM_contrast**	0.04 ± 0.03	0.03	0.00	0.09	0.09
**Metachronous Metastasis Group**					
***Features***	**Mean ± SD**	**Median**	**Min**	**Max**	**Range**
**Volume**	27.76 ± 40.35	7.21	2.86	122.19	119.33
**SUV_max**	5.26 ± 4.88	2.54	1.19	15.14	13.95
**n_voxels**	945.78 ± 1617.10	142.00	81.00	4998.00	4917.00
**Statistic_Energy**	1.01 ± 0.01	0.01	0.01	0.01	0.01
**GLCM_contrast**	0.03 ± 0.03	0.02	0.00	0.10	0.09

**Table 3:** Descriptive statistics of features extracted from MRI images.

**Synchronous Metastasis Group**					
**Features**	**Mean ± SD**	**Median**	**Min**	**Max**	**Range**
**Volume**	9.33 ± 6.40	8.73	1.56	23.30	21.75
**T2_mean**	8.72 ± 8.22	6.58	0.16	24.81	24.65
**GLCM_contrast_T2**	1.87 ± 2.45	0.03	0.00	7.39	7.39
**GLCM_entropy_T2**	1.87 ± 1.72	0.39	0.06	5.47	5.41
**B_value**	5.75 ± 4.71	4.42	0.02	14.54	14.52
**ADC_mean**	5.94 ± 5.06	5.29	0.82	16.23	15.40
**GLCM_contrast_ADC**	1.01 ± 0.02	0.02	0.00	0.07	0.06
**GLCM_entropy_ADC**	1.34 ± 0.06	0.37	0.25	0.40	0.15
**Metachronous Metastasis Group**					
**Features**	**Mean ± SD**	**Median**	**Min**	**Max**	**Range**
**Volume**	17.15 ± 22.04	5.02	2.86	54.53	51.67
**T2_mean**	5.16 ± 5.71	2.09	1.18	15.74	14.56
**GLCM_contrast_T2**	1.01 ± 0.03	0.01	0.00	0.09	0.08
**GLCM_entropy_T2**	1.35 ± 0.03	0.36	0.31	0.41	0.10
**B_value**	4.07 ± 4.14	1.88	1.44	11.58	10.14
**ADC_mean**	2.97 ± 2.94	1.53	0.45	8.50	8.04
**GLCM_contrast_ADC**	1.07 ± 0.15	0.01	0.00	0.41	0.40
**GLCM_entropy_ADC**	0.32 ± 0.13	0.37	0.04	0.38	0.34

## Supplementary Materials

The following are available online at https://www.mdpi.com/2072-6694/11/10/1444/s1: Table S1: Findings evaluated for each BC patient at primary diagnosis.

## Abbreviations

ADC	Apparent Diffusion Coefficient
BC	Breast cancer
DWI	Diffusion Weighted Imaging
ELISA	Enzyme-Linked Immunosorbent Assay
ER	Estrogen Receptor
^18^F-FDG-PET/MRI	Fluorine-18-fluorodeoxyglucose Positron Emission Tomography/ Magnetic Risonance Imaging
GLCM	Gray Level Co-occurrence Matrix
HER2	Human Epidermal Growth Factor Receptor 2
HS	Healthy subjects
IHC	Immunohistochemistry
MM	Metachronous Metastasis
PBMCs	Peripheral blood mononuclear cells
PET/CT	Positron Emission Tomography/ Computed Tomography
PR	Progesteron Receptor
RT-PCR	Reverse Transcription Polymerase Chain Reaction
SM	Synchronous Metastasis
SUV	Standardized Uptake Value
VOI	Volume of interest
YY1	YY1 Transcription Factor
