# Supplementary material for: Hybrid 18F-FDG-PET/MRI Measurement of Standardized Uptake Value Coupled with Yin Yang 1 Signature in Metastatic Breast Cancer. A Preliminary Study
Source: Cancers (Basel). 2019 Sep 26;11(10):1444. doi: 10.3390/cancers11101444 (PMC6827137; doi:10.3390/cancers11101444)
Supplement: Supplementary file 1 [file cancers-11-01444-s001.pdf]

Supplementary
Table 1. Findings evaluated for each BC patient at primary diagnosis

FINDING	INDIVIDUAL										
	**#1**	**#2**	**#3**	**#4**	**#5**	**#6**	**#7**	**#8**	**#9**	**#10**	**#11**
**PET**											
Volume [cm^3]	7,21	5,73	10,29	2,86	23,30	2,90	1,91	7,18	8,73	7,62	4,07
SUV_max [g/ml]	10,16	4,67	2,30	2,27	13,24	8,43	3,78	5,32	7,96	1.33	1,51
n_voxels	204,00	162,00	291,00	81,00	659,00	82,00	54,00	203,00	247,00	119,00	115,00
Statistic_Energy	0,01	0,01	0,01	0,01	0,01	0,01	0,01	0,01	0,01	0,01	0,01
GLCM_contrast	0,06	0,05	0,04	0,01	0,09	0,06	0,03	0,03	0,09	0,03	0,10
**MRI**											
Volume [cm^3]	7,21	8,27	12,34	2,86	23,30	2,90	1,91	7,18	8,73	5,02	4,07
T2_mean	15,74	17,30	1,43	2,09	24,81	10,55	1,41	5,40	12,13	3,55	1,18
GLCM_contrast_T2	0,05	7,39	0,00	0,01	0,08	0,06	0,01	0,02	0,07	0,01	0,09
GLCM_entropy_T2	0,37	5,47	0,32	0,35	0,39	0,41	0,39	0,21	0,41	0,36	0,31
B_value	1,68	10,01	1,58	1,88	14,54	11,58	3,95	0,02	4,42	1,94	8,37
ADC_mean	0,45	9,70	0,82	2,63	16,23	8,50	3,08	5,29	1,17	0,84	5,40
GLCM_contrast_ADC	0,41	0,05	0,00	0,01	0,00	0,04	0,02	0,00	0,07	0,00	0,04
GLCM_entropy_ADC	0,04	0,40	0,31	0,38	0,26	0,37	0,38	0,25	0,40	0,35	0,37
**MOLECULAR DATA**											
YY1 gene expression	5.3	0.15	1.79	7.8	0.83	4.24	0.4	0.59	1.38	4,77	4,01
YY1 serum protein [ng/ml]	1,96	1,71	1,94	1,91	1,96	1,87	2,00	1,67	1,33	1,18	1,97
